# Supplementary material for: Winning and losing in online gambling: Effects on within-session chasing
Source: PLoS One. 2022 Aug 18;17(8):e0273359. doi: 10.1371/journal.pone.0273359 (PMC9387854; doi:10.1371/journal.pone.0273359)
Supplement: S1 File — (PDF) [file pone.0273359.s001.pdf]

Supplemental Materials for *Winning and losing in online gambling: Effects on within-session chasing*

Zhang Chen[1], Roos A. Doekemeijer[1], Xavier Noël[2], and Frederick Verbruggen[1]

[1]Department of Experimental Psychology, Ghent University, Ghent, Belgium

[2]Laboratoire de Psychologie Médicale et d'Addictologie, Faculté de Médecine, Université Libre de Bruxelles, Brussels, Belgium

Author Note

Zhang Chen https://orcid.org/0000-0002-3500-9182

Roos A. Doekemeijer https://orcid.org/0000-0002-5670-2971

Xavier Noël https://orcid.org/0000-0002-4604-8144

Frederick Verbruggen https://orcid.org/0000-0002-7958-0719

Correspondence concerning this article should be addressed to Zhang Chen, Department of Experimental Psychology, Ghent University, Henri Dunantlaan 2, B-9000 Gent, Belgium. Email: zhang.chen@ugent.be

Supplemental Materials for *Winning and losing in online gambling: Effects on within-session chasing*

Contents

Classification of players by Gaming1 **3**

Descriptive information on players and their play behavior **5**

Age and gender distribution 5

Risk levels in the high-involvement group 6

Total number of sessions and rounds played 7

When to stop - without controlling for the overall stopping probabilities **8**

When to stop - redefining 'sessions of play' **11**

Descriptive information 11

Comparisons between groups 13

When to stop 14

Change in stake levels **16**

Speed of play - raw RTs **18**

Classification of players by Gaming1

Gaming1 used several risk indicators to assign players into different risk levels (see Table S1) with an in-house machine learning model. The more risk triggers a player exhibits, the higher risk level a player receives. Note that some risk indicators used by Gaming1 overlap with the diagnostic criteria for gambling disorder in the DSM-5. For instance, the high frequency of play and the escalating deposit or bet amount (Gaming 1 criterion) may overlap with the need to gamble with increasing amounts of money to achieve the desired excitement (DSM-5 criterion); the use of multiple deposit methods (Gaming 1 criterion) may overlap with the reliance on others to provide money (to gamble; DSM-5 criterion); and lastly, the multiple canceled withdrawal or requests for cool-off periods (Gaming 1 criterion) may overlap with (unsuccessful) attempts to control or stop gambling (DSM-5 criterion). Although there are overlaps between the Gaming1 risk indicators and the DSM-5 diagnostic criteria, we acknowledge that the classification scheme by Gaming1 is not a validated measurement of problem gambling. It is therefore unclear whether the gamblers with higher risk levels indeed have greater problem gambling severity. For these reasons, we referred to the two groups as high- vs. low-involvement in the main text.

Table S1

Indicators used by Gaming1 to determine the risk level of a player.

Player Deposit Patterns

Multiple Deposit Methods

- Several different deposit methods during the last 24 hours.
- Several days with multiple deposit methods in the last month.

Player Withdrawal Patterns

Multiple Canceled Withdrawals

- Several canceled withdrawals during the last 24 hours.
- Several days with multiple canceled withdrawals in the last month.

Player Game Control Patterns

Multiple Cool-off Periods

- Several cool-off requests within the last week, or last month.

Player Game Time Patterns

High Frequency of Play

- High number of rounds within the week.

Player Credit Patterns

Deposit Amount Above Average

- The average deposit amount increased significantly in the short term.

Player Game Play Patterns

Important Net Losses

- Important net loss in the last 24 hours or month or year.

Stake Amount Above Average

- The average stake amount of the 5 last playing days is significantly higher than the daily stake average.
- Significant number of high daily stake average in the short term period.

Cumulative Net Gaming

- The cumulative net gaming crosses the lower limit of the band of normal play.

Descriptive information on players and their play behavior

Age and gender distribution

Fig S1 shows the age by gender pyramids for both groups.

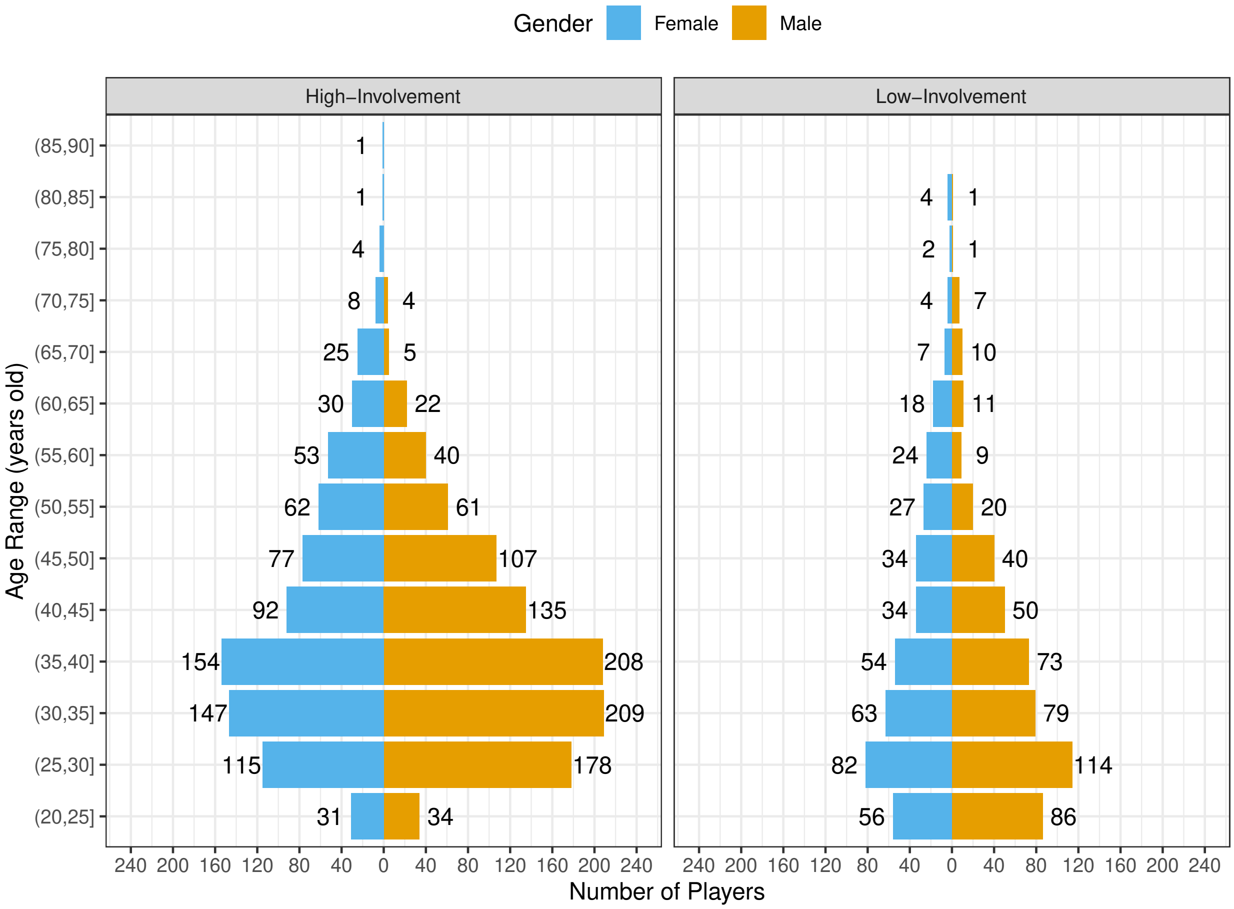

Figure S1

Age by gender pyramids for both groups. *For the y axis, the age range (c, d] includes players who were older than c years old (i.e., >c) but not older than d years old (i.e., ≤ d). The numbers next to the bars indicate the exact numbers of players in each age group.*

Risk levels in the high-involvement group

As noted in the main text, the behavioral indicators of risk level (see Table S1) a player exhibits may vary over time. Accordingly, the risk level of a player can also vary. This is the case for the high-involvement group in the current data (all players in the low-involvement group have consistently a risk level of 0). The Venn diagram below (Figure S2) shows the numbers of players with each risk level in the high-involvement group. Values in the overlapping regions of two or three circles stand for the number of players who have received two or three risk levels over time. For instance, 6 players received a risk level of 3 and 5 over time. Values in the non-overlapping regions stand for the number of players who have received only one risk level. For instance, 108 players received only level 5 in the time period of the current data.

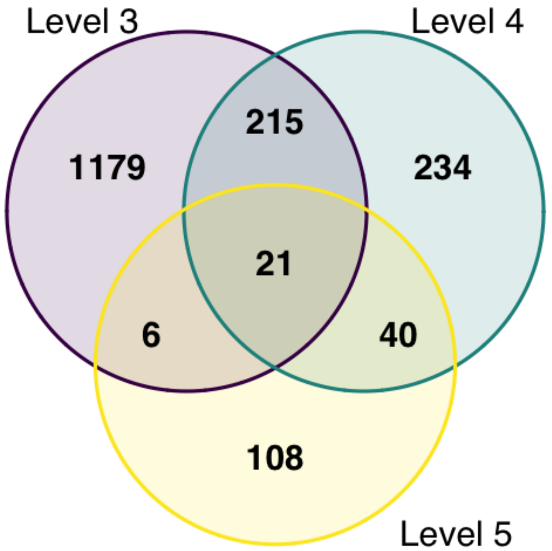

Figure S2

Venn diagram showing the number of players in the high-involvement group with each risk level (determined by Gaming1) in the current data set.

Total number of sessions and rounds played

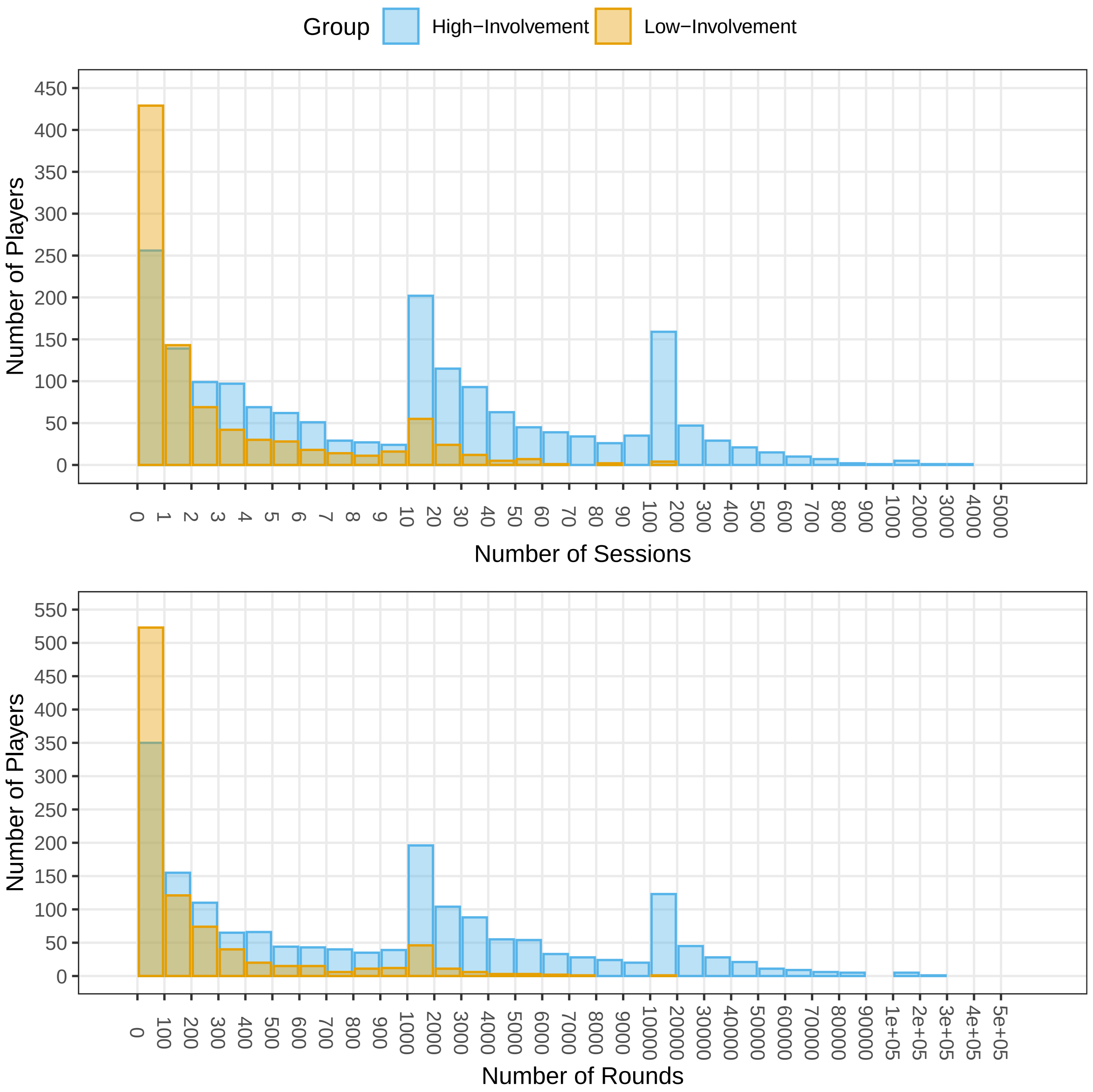

Figure S3

Histograms showing the numbers of players who played certain total numbers of sessions (top) and rounds (bottom). Note that the x axis is transformed. The height of a bar between a and b on the x axis denotes the number of players whose total session or round number is larger than a (> a) but not larger than b (<= b).

When to stop - without controlling for the overall stopping probabilities

Since the high-involvement players tended to have longer sessions, the overall probabilities to end a session was lower in the high-involvement group than in the low-involvement group. In the main text, we controlled for this overall stopping probability by dividing the probability of stopping after a win and after a loss by the overall stopping probability for each player (i.e., p($stop|loss$)/p($stop - overall$) and p($stop|win$)/p($stop - overall$), respectively). Here we present the results based on the raw stopping probabilities (i.e., p($stop|loss$) and p($stop|win$)) for completeness.

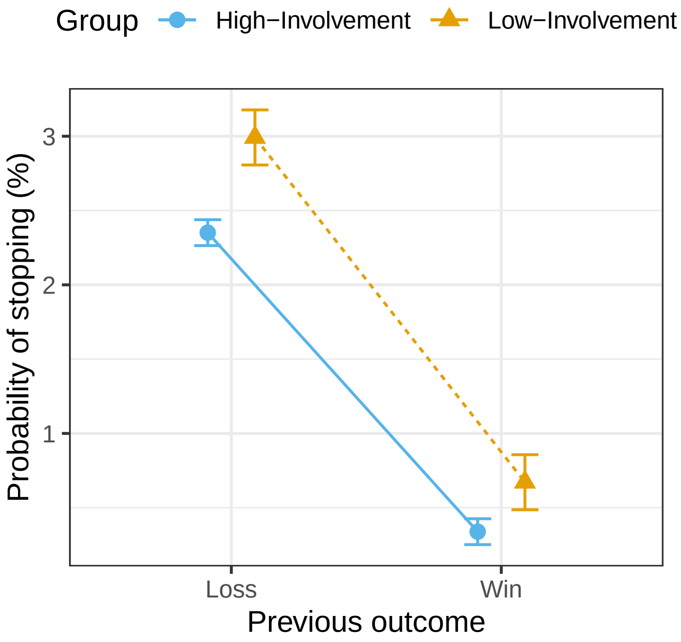

Figure S4

Probability of stopping after a win and after a loss. Error bars stand for 95% within-subject confidence intervals.

The ANOVA on stopping probabilities showed a significant main effect of involvement level, the previous outcome, and their interaction (Table S2). As can be seen in Figure S4, the high-involvement groups indeed had a lower probability to stop after both winning and losing, compared to the low-involvement group. Furthermore, in both groups,

the probability to end a session was higher after a loss than after a win. Consistent with the results reported in the main text, both groups of players showed win-chasing, rather than loss-chasing.

Table S2

Statistical analyses on when to stop (no control for the overall stopping probabilities).

Number of players and rounds included in the analyses in each group

**Group**	**Player**	**Round (Mean)**	**Round (SD)**	**Round (Min)**	**Round (Max)**
High-Involvement	1679	6001.9	14176.8	12	200706
Low-Involvement	651	440.9	959.3	13	13114

ANOVA

**Effect**	**df**	**MSE**	**F**	**ges**	**p**
Involvement Level (High vs. Low)	1, 2328	4.02	55.29	.012	<.001
Previous Outcome (Loss vs. Win)	1, 2328	4.01	1098.91	.191	<.001
Interaction	1, 2328	4.01	5.56	.001	.019

Pairwise comparisons

**Comparison (A vs. B)**	**A-mean**	**B-mean**	**diff**	**lowerCI**	**upperCI**	**df**	**t**	**p**	**lnBF**	**g**
High-Loss vs. High-Win	2.35 (2.24)	0.34 (1.33)	2.01	1.89	2.14	1678.0	32.0	<.001	396.17	1.128
Low-Loss vs. Low-Win	2.99 (2.40)	0.67 (2.35)	2.32	2.06	2.58	650.0	17.4	<.001	120.54	0.977
High-Loss vs. Low-Loss	2.35 (2.24)	2.99 (2.40)	-0.64	-0.85	-0.43	1113.4	-5.9	<.001	15.22	0.272
High-Win vs. Low-Win	0.34 (1.33)	0.67 (2.35)	-0.33	-0.52	-0.14	816.2	3.4	.001	6.19	0.157
(High-Loss - High-Win) vs. (Low-Loss - Low-Win)	2.01 (2.57)	2.32 (3.40)	-0.31	-0.60	-0.02	951.9	-2.1	0.037	-0.21	0.096

Note: *ANOVA*: df = degrees of freedom. In a 2 by 2 ANOVA, the dfs for all effects are the same. MSE = mean square of the error. ges = generalized eta squared. *Pairwise comparisons*: Comparison (A vs. B) = the two variables compared in each comparison. A-mean, B-mean = means of the left (A) and the right (B) variable in a comparison, with standard deviations in parentheses. diff = difference between A and B. lowerCI, upperCI = lower and upper boundary of 95% confidence intervals of the difference. df, t, p = degrees of freedom, t value and p value from the Welch's t tests (between-subjects comparisons) or paired-samples t tests (within-subjects comparisons). P values were corrected for multiple comparisons using the Holm-Bonferroni method. lnBF = the natural logarithm of Bayes factors. g = Hedges's average g.

To break down the interaction effect, we computed a difference score for each player (i.e., p($stop|loss$) - p($stop|win$)). A positive value indicates win-chasing in the facet of when to stop. The difference scores were then compared between the two groups. Contrary to the results in the main text (when the overall stopping probabilities were controlled for), with the raw stopping probabilities, the difference scores were descriptively smaller in the high-involvement group than in the low-involvement group. In other words, the tendency to chase wins seemed to be smaller in the high-involvement players. However, the effect size was rather small, and the Bayes factor was inconclusive (lnBF = -0.21, BF = 0.81).

When to stop - redefining ’sessions of play’

In the main text, we used the operator data to define one ’session of play’. That is, one session is from when a player logged into the platform till when they logged off. Each session received a unique session id in the operator data. However, players may take a break in their play without logging off, which would still be considered as one session in the data. Here, we report extra analyses in which we took such breaks in play (without logging off) into account when defining play sessions.

Players needed to make 13 consecutive responses in each game. If any of the 13 responses took longer than 10 minutes on a particular game, we assume that the player took a break and consider the corresponding game as the first round in a new ’session’ of play. Such breaks in play occurred rather infrequently, in only about 0.050% of the games. We nevertheless still examined whether the results on the decision of when to stop were influenced by this new definition of ’sessions of play’.

Descriptive information

Using this new definition of ’play session’, we counted the number of players who played certain numbers of sessions, and the numbers of sessions that contained certain numbers of rounds. These count numbers are plotted in Figure S5.

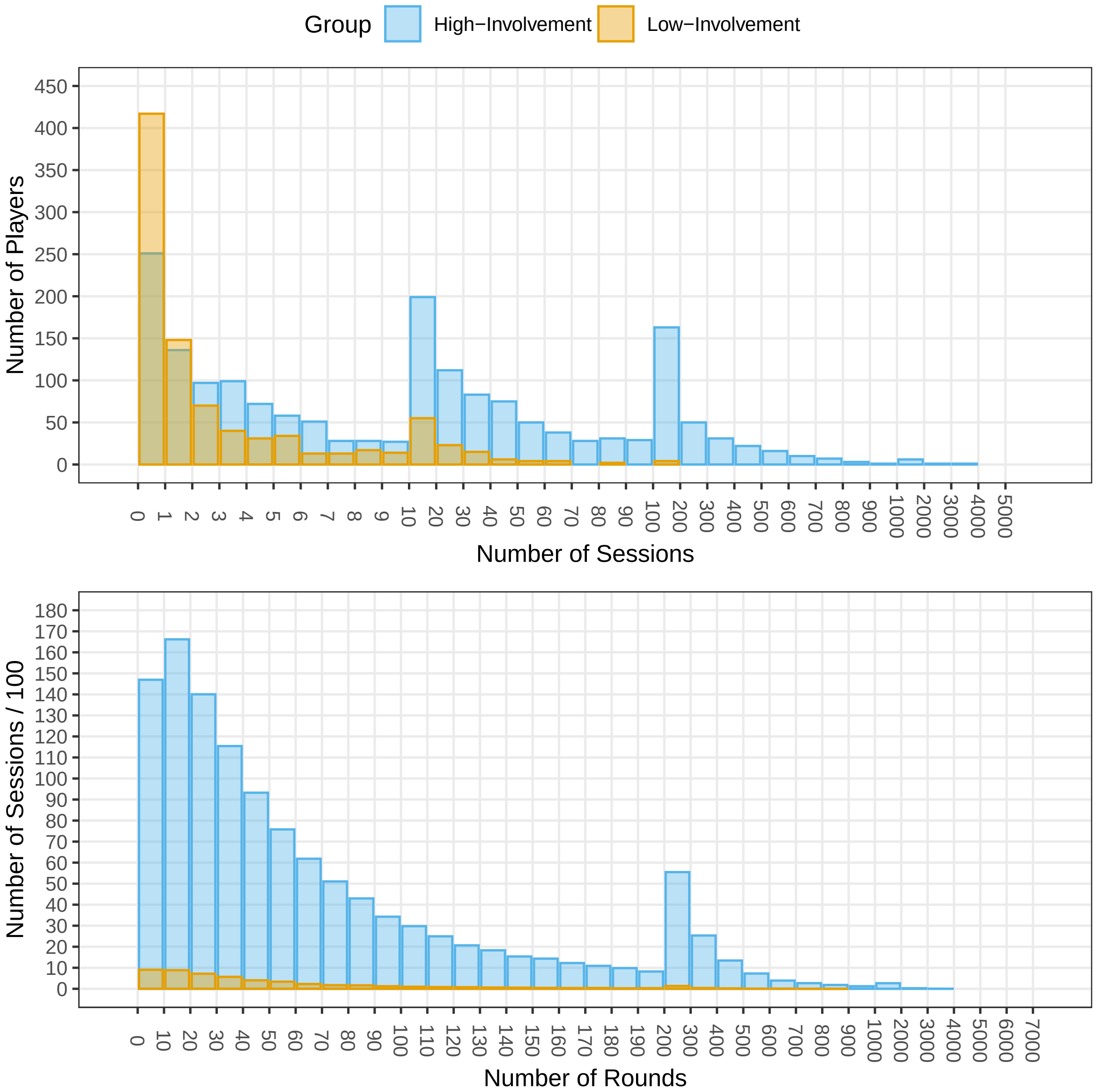

Figure S5

Histograms showing the numbers of players who played certain numbers of sessions (top) and the numbers of sessions that contained certain numbers of rounds (bottom; players from the same group were combined). Note the x axis is transformed. The height of a bar between a and b denotes the number of players (or the number of sessions / 100) whose session number (or round number) is larger than a (> a) but not larger than b (<= b).

Comparisons between groups

Table S3

Comparing number of sessions and rounds between the two groups

Parameter	High	Low	diff	lowerCI	upperCI	df	t	p	lnBF	g
Session Number	66.9 (168)	5.8 (12.6)	61.1	53.3	69.0	1842.2	15.4	<.001	55.4	0.625
Mean Round Number	77.8 (68.3)	49.8 (61.4)	28.0	22.9	33.1	2004.4	10.8	<.001	49.8	0.438
Median Round Number	57.8 (58.7)	44.8 (59.7)	13.0	8.3	17.8	1796.0	5.4	<.001	11.5	0.219

Note: High, Low = means of parameters for two groups, with standard deviations in parentheses. diff = difference between the high-involvement group and the low-involvement group. lowerCI, upperCI = lower and upper boundary of 95% confidence intervals of the difference. df, t, p = degrees of freedom, t value and p value from the Welch's t-tests. P values were corrected for multiple comparisons using the Holm-Bonferroni method. lnBF = the natural logarithm of Bayes factors. g = Hedges's g.

Next we compared the number of sessions, the mean and median number of rounds played in a session between the high- and the low-involvement group. The results can be found in Table S3. As would be expected, compared to the corresponding table in the main text, the average number of sessions played by players *increased*, while the average number of rounds per session (both the means and the medians) *decreased*, as the sessions in the original data have been divided into multiple shorter sessions. Importantly, the difference between the two groups remained: the high-involvement players overall played more sessions, and their play sessions also tended to be longer (i.e., containing more rounds).

When to stop

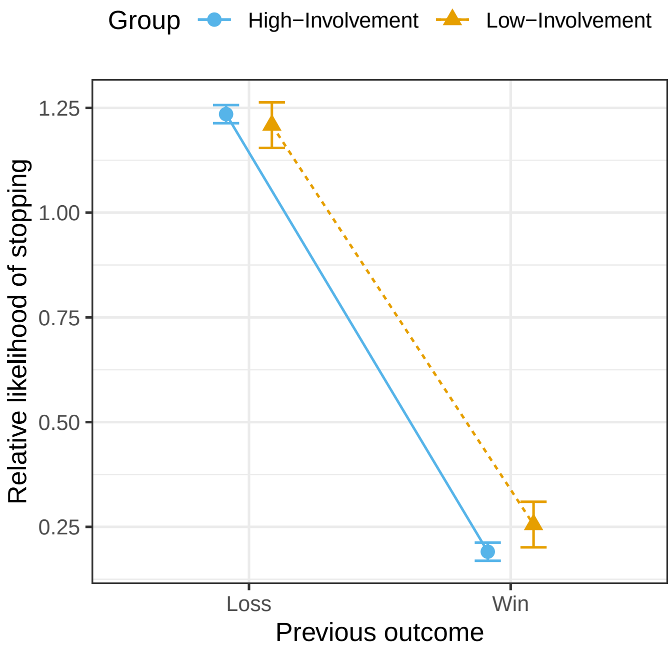

Figure S6

Probability of stopping after a win and after a loss. Error bars stand for 95% within-subject confidence intervals.

We repeated the same analyses on the decision of when to stop, by controlling for the overall probability to end a session for each player. The overall pattern of the results remained the same (Figure S6 and Table S4). Again, both groups of players were more likely to stop playing the game after a loss than after a win. Although the tendency to chase wins was descriptively larger in the high-risk group than in the low-risk group, the size of this effect was rather small, and the Bayes factor was inconclusive (lnBF = 0.39, BF = 1.48).

Table S4

Statistical analyses on when to stop (taking breaks longer than 10 minutes into account).

Number of players and rounds included in the analyses in each group					
**Group**	**Player**	**Round (Mean)**	**Round (SD)**	**Round (Min)**	**Round (Max)**
High-Involvement	1679	6001.9	14176.8	12	200706
Low-Involvement	651	440.9	959.3	13	13114

ANOVA					
**Effect**	**df**	**MSE**	**F**	**ges**	**p**
Involvement Level (High vs. Low)	1, 2328	0.09	3.69	<.001	.055
Previous Outcome (Loss vs. Win)	1, 2328	0.29	3259.61	.513	<.001
Interaction	1, 2328	0.29	6.75	.002	.009

Pairwise comparisons										
**Comparison (A vs. B)**	**A-mean**	**B-mean**	**diff**	**lowerCI**	**upperCI**	**df**	**t**	**p**	**lnBF**	**g**
High-Loss vs. High-Win	1.24 (0.15)	0.19 (0.49)	1.04	1.01	1.07	1678.0	66.8	<.001	1083.78	3.215
Low-Loss vs. Low-Win	1.21 (0.22)	0.26 (0.79)	0.95	0.88	1.03	650.0	24.4	<.001	206.85	1.880
High-Loss vs. Low-Loss	1.24 (0.15)	1.21 (0.22)	0.03	0.01	0.04	901.0	2.7	0.019	2.13	0.126
High-Win vs. Low-Win	0.19 (0.49)	0.26 (0.79)	-0.06	-0.13	0.00	855.2	-1.9	0.063	-0.18	0.090
(High-Loss - High-Win) vs. (Low-Loss - Low-Win)	1.04 (0.64)	0.95 (1.00)	0.09	0.01	0.17	865.2	2.2	0.063	0.39	0.100

Note: *ANOVA*: df = degrees of freedom. In a 2 by 2 ANOVA, the dfs for all effects are the same. MSE = mean square of the error. ges = generalized eta squared. *Pairwise comparisons*: Comparison (A vs. B) = the two variables compared in each comparison. A-mean, B-mean = means of the left (A) and the right (B) variable in a comparison, with standard deviations in parentheses. diff = difference between A and B. lowerCI, upperCI = lower and upper boundary of 95% confidence intervals of the difference. df, t, p = degrees of freedom, t value and p value from the Welch's t tests (between-subjects comparisons) or paired-samples t tests (within-subjects comparisons). P values were corrected for multiple comparisons using the Holm-Bonferroni method. lnBF = the natural logarithm of Bayes factors. g = Hedges's average g.

Change in stake levels

In the game, players could choose one out of ten stake amounts (0.25, 0.50, 1.00, 1.50, 2.00, 2.50, 5.00, 10.00, 15.00 and 20.00 euro, respectively). In the main text, we focused on the change in stake amount (euro cents) as a function of previous outcome. Since the increment between two consecutive stake levels was not always the same, we additionally analyzed the change in stake *levels*. The ten stake amounts were first converted into stake levels, with 1 being the lowest stake of 0.25 euro and 10 being the highest stake of 20.00 euro. We then calculated the average change in stake level between two games. Since players overall changed the stakes rather infrequently, the average changes in stake level were quite small. To reduce the number of leading zeros, we therefore multiplied the changes in stake level by 100. The changes in stake level were then analyzed, using the same data analysis methods as in the main text (Figure S7).

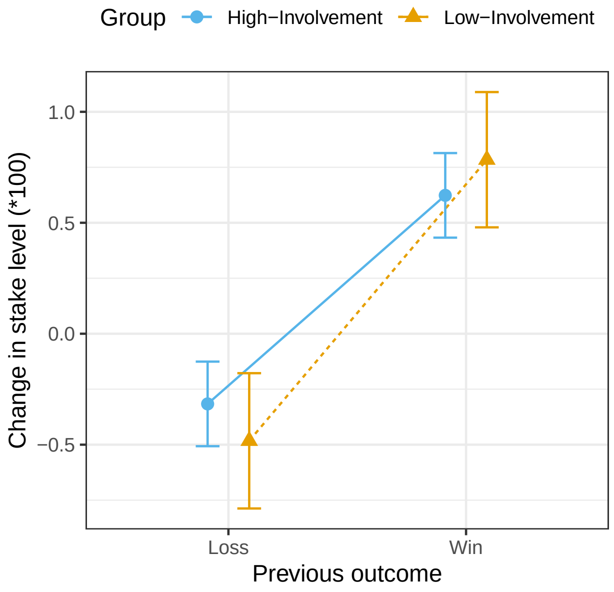

Figure S7

*Change in stake levels (*100) after a win and after a loss. Error bars stand for 95% within-subject confidence intervals.*

The results were largely in line with those obtained with the changes in stake

Table S5

Statistical analyses on change in stake level.

Number of players and rounds included in the analyses in each group

Group	Player	Round (Mean)	Round (SD)	Round (Min)	Round (Max)
High-Involvement	1678	5936.6	14046.5	11	197613
Low-Involvement	648	435.5	949.0	13	12959

Change in stake level (* 100) - ANOVA

Effect	df	MSE	F	ges	p
Involvement Level (High vs. Low)	1, 2324	6.38	0.00	<.001	.973
Prior Outcome (Loss vs. Win)	1, 2324	15.78	72.12	.022	<.001
Interaction	1, 2324	15.78	1.58	<.001	.208

Change in stake level (* 100) - Pairwise comparisons

Comparison (A vs. B)	A-mean	B-mean	diff	lowerCI	upperCI	df	t	p	lnBF	g
High-Loss vs. High-Win	-0.32 (2.38)	0.62 (4.05)	-0.94	-1.21	-0.67	1677.0	-6.8	<.001	19.43	0.292
Low-Loss vs. Low-Win	-0.48 (2.62)	0.78 (3.94)	-1.27	-1.70	-0.84	647.0	-5.8	<.001	13.05	0.386
High-Loss vs. Low-Loss	-0.32 (2.38)	-0.48 (2.62)	0.17	-0.06	0.40	1085.5	1.4	1.000	-1.89	0.065
High-Win vs. Low-Win	0.62 (4.05)	0.78 (3.94)	-0.16	-0.52	0.20	1204.0	-0.9	1.000	-2.59	0.040
(High-Loss - High-Win) vs. (Low-Loss - Low-Win)	-0.94 (5.63)	-1.27 (5.59)	0.33	-0.18	0.84	1183.3	1.3	1.000	-2.17	0.058

Note: *ANOVA*: df = degrees of freedom. In a 2 by 2 ANOVA, the dfs for all effects are the same. MSE = mean square of the error. ges = generalized eta squared. *Pairwise comparisons*: Comparison (A vs. B) = the two variables compared in each comparison. A-mean, B-mean = means of the left (A) and the right (B) variable in a comparison, with standard deviations in parentheses. diff = difference between A and B. lowerCI, upperCI = lower and upper boundary of 95% confidence intervals of the difference. df, t, p = degrees of freedom, t value and p value from the Welch's t tests (between-subjects comparisons) or paired-samples t tests (within-subjects comparisons). P values were corrected for multiple comparisons using the Holm-Bonferroni method. lnBF = the natural logarithm of Bayes factors. g = Hedges's average g.

amount (Table S5). There was again a significant main effect of prior outcome, such that players increased the stake level more after a win than after a loss. The only difference was that this effect was statistically reliable in both groups for changes in stake *levels*, whereas for changes in stake *amount*, the effect was only statistically reliable in the high-involvement group (see the main text).

Speed of play - raw RTs

In the main text, we used RT z scores as an index for the speed of play, to control for the general difference in the speed of play across players. Here we analyzed the raw RT scores (from starting a game till putting in the first column, in milliseconds) with the same data analysis method.

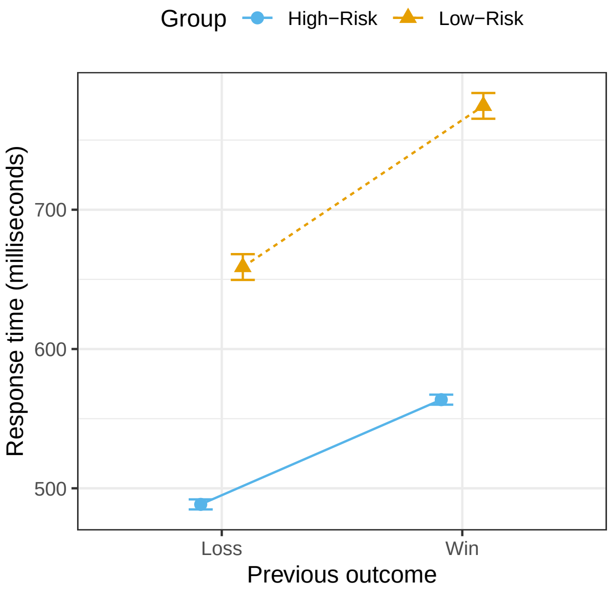

Figure S8

Speed of play (raw RT values in milliseconds) after a win and after a loss. Error bars stand for 95% within-subject confidence intervals.

High-involvement players overall played more quickly than low-involvement players (Figure S8 and Table S6). The main effect of prior outcome was also statistically significant; players played a game more quickly after a loss than after a win, and this effect was observed for both groups of players (see the pairwise comparisons in Table S6). Furthermore, there was a significant interaction effect between involvement level and prior outcome. To break down this interaction effect, for each player, we calculated a difference score between RT after a loss and RT after a win (i.e., $RT_{diff} = RT_{loss}$ - RT_{win}). These difference scores were then compared between the high-involvement and the

Table S6

Statistical analyses on speed of play, using raw RT values (in milliseconds).

Number of players and rounds included in the analyses in each group

Group	Player	Round (Mean)	Round (SD)	Round (Min)	Round (Max)
High-Involvement	1678	5914.4	13975.3	11	196747
Low-Involvement	646	433.3	943.7	16	12856

ANOVA

Effect	df	MSE	F	ges	p
Involvement Level (High vs. Low)	1, 2322	123081.68	275.54	.100	<.001
Prior Outcome (Loss vs. Win)	1, 2322	8066.72	1053.60	.027	<.001
Interaction	1, 2322	8066.72	47.47	.001	<.001

Pairwise comparisons

Comparison (A vs. B)	A-mean	B-mean	diff	lowerCI	upperCI	df	t	p	lnBF	g
High-Loss vs. High-Win	488.4 (202.0)	563.6 (238.5)	-75.2	-80.3	-70.1	1677.0	-28.9	<.001	335.49	0.341
Low-Loss vs. Low-Win	658.9 (300.8)	774.6 (357.3)	-115.7	-128.8	-102.6	645.0	-17.4	<.001	120.34	0.351
High-Loss vs. Low-Loss	488.4 (202.0)	658.9 (300.8)	-170.4	-195.6	-145.2	878.3	-13.3	<.001	114.20	0.615
High-Win vs. High-Loss	563.6 (238.5)	774.6 (357.3)	-210.9	-240.8	-181.1	875.4	-13.9	<.001	124.56	0.642
(High-Loss - High-Win) vs. (Low-Loss - Low-Win)	-75.2 (106.4)	-115.7 (169.2)	40.5	26.5	54.5	849.0	5.7	<.001	20.37	0.262

Note: *ANOVA*: df = degrees of freedom. In a 2 by 2 ANOVA, the dfs for all effects are the same. MSE = mean square of the error. ges = generalized eta squared. *Pairwise comparisons*: Comparison (A vs. B) = the two variables compared in each comparison. A-mean, B-mean = means of the left (A) and the right (B) variable in a comparison, with standard deviations in parentheses. diff = difference between A and B. lowerCI, upperCI = lower and upper boundary of 95% confidence intervals of the difference. df, t, p = degrees of freedom, t value and p value from the Welch's t tests (between-subjects comparisons) or paired-samples t tests (within-subjects comparisons). P values were corrected for multiple comparisons using the Holm-Bonferroni method. lnBF = the natural logarithm of Bayes factors. g = Hedges's average g.

low-involvement group. We observed that wins and losses had a smaller influence on the speed of play for high-involvement players than for low-involvement players. However, since the high-involvement players overall played the games much more quickly, this smaller influence of prior outcomes could be (partly) caused by this general difference in the speed of play between the two groups. We therefore reported the analyses with RT z scores in the main text, but included the results on raw RT values here for the sake of completeness.
