# Supplementary material for: Winning and losing in online gambling: Effects on within-session chasing
Source: PLoS One. 2022 Aug 18;17(8):e0273359. doi: 10.1371/journal.pone.0273359 (PMC9387854; doi:10.1371/journal.pone.0273359)
Supplement: Attachment — Submitted filename: Plos-One-Revision.pdf [file pone.0273359.s002.pdf]

Dear editor,

Thank you for the opportunity to revise our manuscript, *Winning and losing in online gambling: Effects on within-session chasing*. On behalf of my co-authors, I would like to thank you and the two reviewers for your invaluable comments on our manuscript. We think our manuscript is substantially improved after addressing the comments and making the suggested edits.

In this letter, we list the comments by you and the two reviewers **in bold**, and explain how we have addressed each comment in the revision. Texts copied and pasted from the revised manuscript are shown in green. In the pdf file of the revised manuscript, we have highlighted the major changes.

Kind regards,
Zhang Chen

Comments by the editor

1. On the overall, the introduction and discussion are well written, but the material and statistical analysis need to be reworked. That's one of the main issues of this article. The methods and statistical analysis seem to have been correctly done but the way it is explained is far too complex. In addition, the discussion lacks perspective and there is no clear message regarding the results of this study under the scope of the current literature.

Thank you for these suggestions. To make the methods and the statistical analyses easier to follow, we have restructured these parts by using subsections, and by always starting with the most important general information (please also see our reply to Comment 3 below for how we restructured the method section).

For the statistical analyses section, we start with an overview of the analyses on loss-chasing:

We conducted three sets of analyses, each focusing on one facet of loss-chasing. Table 2 provides an overview of the behavioral indicator used, the main analysis, the expression of loss-chasing, and (as a preview of the results) whether loss-chasing was observed or not for each facet. In the sections that follow, we explain each set of analyses in detail.

Table 2. An overview of the behavioral indicator, main analysis, loss-chasing expression and whether it was observed in the current study for each facet.

Facet	Behavioral indicator	Main analysis	Loss-chasing expression	Observed?
When to stop	The probabilities of ending a session after winning and losing, while controlling for the overall probability of stopping.	Mixed ANOVA on the relative likelihoods of stopping, with the prior outcome (loss vs. win, within-subjects) and involvement level (high vs. low, between-subjects) as factors.	The probability of ending a session will be lower after a loss than after a win.	No
Change in stake	The probability of changing the stake, and the average change in stake amount after winning and losing.	Mixed ANOVA on the probability of changing stake and the average change in stake amount, with the prior outcome (loss vs. win, within-subjects) and involvement level (high vs. low, between-subjects) as factors.	Players will increase the stake amount more after a loss than after a win.	No
Speed of play	How quickly players place the first column of dice after starting a round (z score) after winning and losing.	Mixed ANOVA on the mean RT z scores, with the prior outcome (loss vs. win, within-subjects) and involvement level (high vs. low, between-subjects) as factors.	Players will place the first column of a round more quickly after a loss than after a win.	Yes

For the analyses of each facet, we then always used the same structure: first the aim of the analyses (i.e., the Aim of analyses subsection), then the specific behavioral indicator selected and the motivation for selecting the specific indicator (i.e., the Behavioral indicator subsection), and lastly the statistical analyses used (i.e., the Analysis methods subsection). By using this structure, we think the statistical analyses will now be easier to follow.

Furthermore, we agree that in the original manuscript, the analysis on when to stop was too difficult and confusing (as both reviewers commented on this particular facet as a major point). We have now adjusted the analyses of this facet. We think that the new analysis is more intuitive. And as a result, the same analysis approach is now used for all three facets. By bringing more consistency to the analysis approach across the facets, we think the overall analysis and result sections have become much clearer.

Lastly, concerning the discussion of the results under the scope of the current literature, we agree that the original manuscript only discussed the three facets in isolation, and did not go further in discussing what these findings mean for chasing in real gambling more generally. We have now done that in the revised manuscript, by adding in the general discussion a new section "Chasing: A multifaceted phenomenon".

One strength of the current work is the simultaneous examination of three behavioral expressions of within-session loss-chasing. Among the three facets examined, we observed win-chasing in (1) when to stop and (2) change in stake amount, and loss-chasing in (3) the speed of play. Instead of chasing wins or losses in all aspects of gambling behavior, gamblers seemed to intensify some facets after winning, and other facets after losing. This finding highlights the multifaceted and dynamic nature of chasing. To understand chasing, it is therefore necessary to simultaneously characterize multiple aspects of gambling behavior. We note that the speed of play may be an important facet via which chasing can be expressed. As discussed above, after losing, gamblers may not have enough money to place a larger stake, and might even be forced to stop. These factors may contribute to the observed win-chasing in the first two facets. By contrast, as long as gamblers have sufficient funds to continue playing, the speed of play is not limited by prior outcomes. As such, the speed of play may serve as a 'pure' measure of gambler's urge to continue playing, since it

is not constrained by available funds. The speed of play has received less attention than the other two facets so far. Our results suggest that it might be fruitful to consider the speed of play when examining chasing in real gambling.

Wins and losses had comparable effects in both the low- and high-involvement groups. Overall, we did not observe a stronger tendency to chase losses in players who were more involved in gambling. This finding seems to run counter to the idea that loss-chasing is a defining feature of problem gambling [6], and may thus be more pronounced as people become more involved in gambling. One possibility is that gambling involvement based on operator tracking data may not correlate with problem gambling severity (see below). Second, the clinical diagnosis of gambling disorder (e.g., DSM-5 [4]) uses between-session chasing as a criterion, while we examined within-session chasing here. On the one hand, within-session chasing can be a building block for between-session chasing [8]. For instance, extensive and intensified gambling within a session may be more likely to lead to substantial financial losses (due to the negative expected value of most gambling products), which may lead gamblers to come back another time to try to recoup previous losses (i.e., between-session chasing). On the other hand, between-session chasing does not necessarily mean that loss-chasing is also intensified within a session. Indeed, although the highly involved players gambled more intensively, wins and losses in the previous round had a smaller influence on their speed of play. This potential dissociation between within- and between-session chasing again highlights the multifaceted nature of loss-chasing, and the necessity of carefully delineating different facets of chasing in real gambling.

2. Introduction: Hypothesis: It seems there was no clear hypothesis, and it was more an exploratory study. I would appreciate further explanation on this matter.

Before conducting the analyses, we indeed did not have directional predictions for each of the facets (thus we also did not pre-register any hypotheses). We now comment explicitly on this aspect in the introduction.

Given the multifaceted and dynamic nature of loss-chasing and some inconsistent findings in the literature, *a priori* we did not have strong predictions for whether loss-chasing would occur in each facet or not. This study was therefore exploratory in nature, and mainly aimed at characterizing loss-chasing (i.e., its presence or absence in each facet) in real online gambling.

3. Material and methods: The game is extremely simple but when referring to the figure and the explanation of the game gave by the authors, this game seemed too complex. I think the authors need to improve the figure 1 (also please place figure 1 under the figure), and the game explanation. Figure 1 should allow reader to understand how the game works at first sight. Essential information could be provided first, followed by the additional information for further details.

In the Procedure section, we first explain the general rules of the game before discussing the game interface in detail.

Before starting a round, players needed to place a stake (Fig 1). The stake was for the whole round, and could not be changed after pressing start. Twelve columns of dice (three dice per

column) then appeared one by one. Players needed to put these 12 columns into 4 slots, with 3 columns in each slot. They won points in a slot when a horizontal or a diagonal line of the slot contained the same dice. After all 12 columns had been placed, the points from all 4 slots were added up and converted into monetary prizes (win or loss). After finishing a round, players could continue or end the session.

Furthermore, we have added a new Figure 1 that depicts the events within one round, and on which events each facet of loss-chasing is based on in the current study.

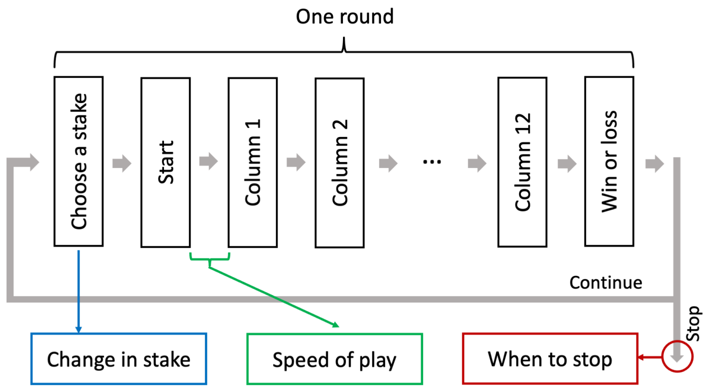

Figure 1. A flowchart showing the events within one round, and on which event each expression of loss-chasing is based.

To make sure that readers can understand the game based on Figure 2 (originally Figure 1), we have now added the general rules to the figure caption, and placed the caption beneath the figure.

4. Measures: Same as previous comments, please provide essential information first and then provide details. A summarizing table with measures, tests and associated hypothesis would be appreciated.

Please see our reply to Comment 1 above, and Table 2 for a summarizing table with behavioral indicators, tests, loss-chasing expressions and observed results for each facet.

In the Measures section, we now list the variables in the behavioral tracking data and their explanations in Table 1 to improve the readability.

Table 1. An overview of the variables in the behavioral tracking data.

Variable	Explanation
Player ID	A unique, deidentified ID for each player.
Session ID	A unique, deidentified ID for each session. One session consists of all rounds from when a player logged into the game, till when they disconnected.
Round ID	A unique, deidentified ID for each round. One round consists of placing all 12 columns of dice into the 4 slots.
Stake amount	The stake amount in each round, in euro.
Win amount	The amount of money a player won in a round, in euro. In case of a loss, the win amount is 0.
Speed of starting a round	The duration from when players placed the last column in the previous round, till when they started the current round, in milliseconds.
Speed of placing the columns	How quickly players placed each of the 12 columns into the slots, in milliseconds. Note that we used the speed of placing the first column as the behavioral indicator of speed of play.
Bonus game	Whether a bonus game occurred or not in a round.

5. Statistical analysis: The presentation of the statistical analysis is too complex, and readers will get lost very quickly. The authors need to provide the purpose of each of the analyses so the readers will understand easily. There are several other issues such as: Line 235, nothing is specified about the two groups; Line 250 how the High-involvement and low-involvement players group are defined? I couldn't find anything in the method.

Please see our reply to Comment 1 above to see how we used this strategy to improve the structure and presentation of the statistical analyses.

To make the definition of the two groups more salient, we have now divided the Measures section into two subsections, with the second subsection titled Defining the low- and high-involvement groups.

Defining the low- and high-involvement groups

The operator also tracks each player's overall monetary and time expenditure on their platform (see the Supplemental Materials for a detailed description of the indicators). Based on these indicators, each player is assigned a level of gambling involvement from the operator, from 0 to 5, with a higher level standing for higher involvement. Gaming1 provided us with data from players with levels 0, 3, 4, and 5, but no data from players with levels 1 and 2. Since the indicators that players exhibit may vary over time, the involvement level of a player can accordingly also change. This is the case for some level 3/4/5 players in the data, where one player had different involvement levels across time. All level 0 players in the data had consistently level 0. Because the involvement level varied within some players, and that we do not have data on which indicators each player exhibited (i.e., Gaming1 only provided us general scores), we therefore focused on the comparison between the level 0 players and the level 3/4/5 players, without making further distinctions among levels 3, 4 and 5. We will refer to the level 0 players as the low-involvement group, and the level 3/4/5 players as the high-involvement group.

Furthermore, when we first mention the two groups in the *Statistical analysis* section, we refer the readers back to the previous section.

To examine whether and to what extent the two groups differed in their gambling involvement, we conducted a series of comparisons between the high- and low-involvement groups (see the 'Defining the low- and high-involvement groups' section above for how the two groups are defined).

6. Results Figure 3 is misleading with the shifted lines between groups and the vertical reading arrangement. In addition, I do not understand why the three behavioral expressions are presented within the same figure. The arrangement of the figure should be based on a horizontal reading pattern with A and B as the top left and top right sub figure and the C as the bottom left and bottom right sub figure.

We decided to present the results of all three behavioral expressions together, because the main aim of the current paper is to characterize all three facets in a single online commercial game. It would thus be useful to present all facets in one plot, to show the presence/absence of loss-chasing in each facet.

We have changed the arrangement of the sub-plots to conform to a horizontal reading pattern, and in the same order as in the Results section. Furthermore, we tried to reduce the horizontal shift between the two groups as much as possible. However, it is necessary to dodge the lines side by side, otherwise the points and the error bars of the two groups would be overlapping, making the plots difficult to read. Please see the updated figure below.

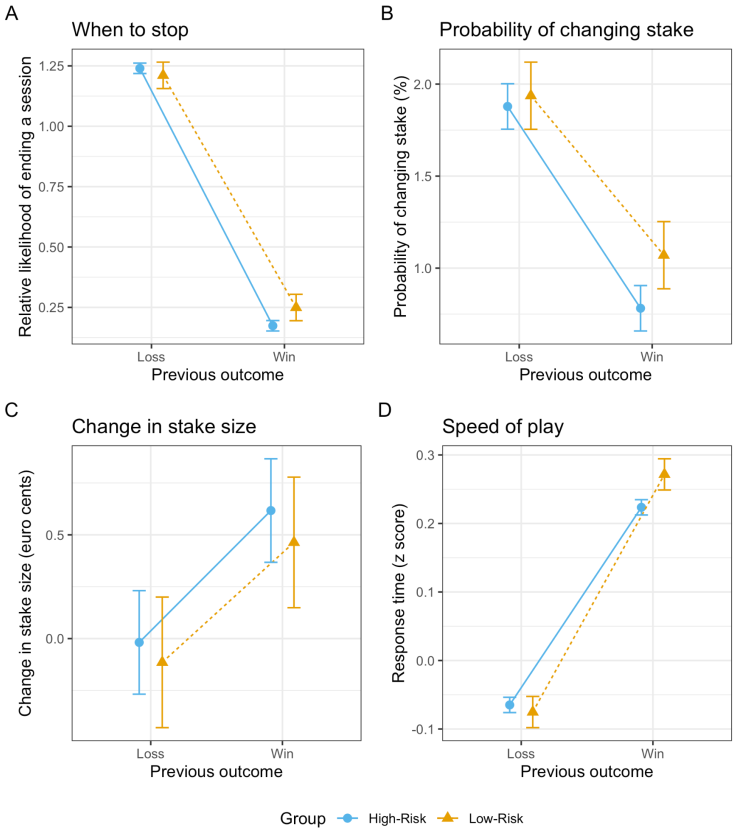

Fig 3. Behavioral expressions of within-session chasing. (A) when to stop, (B) probability of changing stake, (C) change in stake size, and (D) speed of play. Error bars stand for 95% within-subject confidence intervals.

Comments by reviewer 1

1. I have reviewed this manuscript previously – and positively – for a different journal, and I would like to say what a pleasant surprise it is to see that most of my previous concerns have been addressed in this new submission.

This is an unusual and sophisticated piece of research using naturalistic gambling data from a specific online gambling game (‘Mystery Arena’) in a large sample (over 2500 gamblers making over 10 million bets). The paper provides a detailed behavioural analysis of ‘loss chasing’, a key feature of problematic gambling. An important contribution is the description of 3 distinct expressions of loss chasing: ‘when to stop’ (linked to persistence), the amount bet, and the speed of play. They compare these variables between winning and losing gambles, and between groups classified by the gambling operator as higher or lower risk, using their own (proprietary) algorithm. The indicators used by the gambling operator are shown in the Supp material, and while this classification is a little opaque (given the proprietary aspect), there is certainly useful information in these analyses, and I thought the authors handled its (limited) validity very appropriately. The groups differ primarily on the stopping variable (although not in the conventional ‘loss’ chasing direction) and on the speed of play variable.

Thank you for your kind words, and for your previous comments, which have already been invaluable in helping us improve the manuscript.

2. As with much research, a key strength is also a key weakness: the authors focus on Mystery Arena as one specific gambling product, because chasing may be expressed differently on different kinds of gambling (e.g. slot machine gamblers may persist whereas sports bettors may escalate their stake size). The authors also note that it is better suited to the speed of play analysis that a conventional slots game. I fully agree on both accounts, but I might add as a limitation that it seems a slightly unorthodox game. The authors refer on pg 16 that it is ‘chance based’ and compare on pg 4 to a ‘conventional slot machine’. I would say that the behavioural requirements per bet (‘skill’, in a colloquial sense) are greater than a slot machine, and in the manipulation checks, the high involvement do seem to win more than the low involvement groups. To what extent do the motor requirements of the game actually afford experienced players control over the return to player? Could the authors add a clear statement (perhaps in the task methods) as to where this game falls on the skill /chance continuum? Later on in the manuscript, they also refer to the game as a “gamble” e.g. Discussion line 1. I would suggest term ‘gambling product’.

Thank you for this comment. We agree that *Mystery Arena* contains more 'skill' components than conventional slot machines, which may explain why the high-involvement group overall had a higher chance of winning compared to the low-involvement group - a possibility that we did not consider previously. We have now added a paragraph on where we think this particular gambling product falls on the skill/chance continuum when describing the general rules of the game.

Gambling products are often categorized into two broad types, chance-based games that involve little deliberation or skill (e.g., slot machines, lotteries), and skill-based games in which players can exert some influence over the outcome (e.g., Blackjack, sports betting) [31]. On the continuum from chance-based to skill-based games, *Mystery Arena* sits between the two endpoints, but probably somewhere closer to the endpoint of chance-based games. That is, the dice are randomly generated, which largely determines the outcome (i.e., the chance component). However, players can decide in which slot to place each column, which also influences the outcome (i.e., the skill component).

When describing the between-group difference in winning probabilities, we now add the possibility that high-involvement players may be more 'skilled' than low-involvement players.

The overall probability of winning was higher in the high- than the low-involvement group. Two not mutually exclusive explanations exist. First, some players might play relatively few games, and decide to stop after experiencing a low winning probability. These players were more likely to be classified into the low-involvement group. Second, the game involved a skill component, as players needed to decide where to place each column. The high-involvement players might have a better knowledge of how to place the columns, and therefore achieved higher winning probabilities.

We now use the term 'gambling product' throughout the whole manuscript.

3. Of the 3 definitions of chasing, I feel the 'when to stop' analysis is the most challenging; for the change in stake and speed of play analyses, I feel there would be much agreement among researchers about how to operationalize those variables. For 'when to stop', the authors approach this in an interesting way, by looking at the probability of winning for that player's overall 'session', and the pwin on their last trial (i.e. before they stop). As p(win-end) is less than p(win-overall), they conclude that players stop when they are losing, i.e. the opposite of loss 'chasing'. This is the first paper that I have seen operationalize chasing in this way, and it's an interesting way of simplifying a very complex variable. At the same time, it loses some of the dynamics of chasing, by ignoring the length of the session, and it doesn't seem to adequately orthogonalize the responses to winning and losing. For example, the authors acknowledge that their effect (the low pwin-end) could reflect stopping after a losing streak, or bigger wins early in the session, and/or exhausting funds. The secondary analysis excluding breaks over 10 minutes does not really shed any light on these ambiguities. Overall, I think the authors could stick with this analysis, but I would encourage them to note that there are other ways to operationalize this variable, e.g. the logistic regression by Leino.

Thank you for this important comment. We realized that our initial operationalization might indeed be uncommon and difficult to follow (as Reviewer 2 also had a major comment on this issue). To simplify the analyses and the interpretation of the results, we therefore decided to use a different approach that still controlled for the overall probability of stopping. More specifically, for the analyses on when to stop:

Aim of analyses The first set of analyses focused on whether winning and losing would influence the probability of ending a session, as an expression of loss-chasing.

Behavioral indicator We used the probabilities of ending a session after a win and after a loss as the behavioral indicators of when to stop. More concretely, for each player, we computed the total number of winning rounds (all sessions combined), and the number of winning rounds that were at the end of a session (i.e., players decided to stop after these rounds). Dividing the latter by the former resulted in the probability of stopping after winning (i.e., p(stop|win)). The probability of stopping after losing (i.e., p(stop|loss)) was then similarly computed. If players chased losses by continuing playing, p(stop|loss) would be lower than p(stop|win). Note that this analysis is conceptually similar to the logistic regression used by Leino et al. [9], who examined the effects of gambling outcomes on the decision to continue vs. stop as a binary variable.

Since the high-involvement players on average had longer sessions (as a criterion to create the two groups), the overall probability of ending a session was lower for the high-involvement players than for the low-involvement players (see the Supplemental Materials). We therefore sought to control for the overall probability of stopping in the analyses. For each player, we computed the total number of rounds (all sessions combined, regardless of the outcome) and the number of rounds at the end of a session. Dividing the latter by the former resulted in the overall probability of stopping (i.e., p(stop-overall)). For each player, we divided both p(stop|win) and p(stop|loss) by p(stop-overall). The resulting values indicated the relative likelihoods of stopping after winning and losing, controlling for each player's general probability of stopping.

Analysis methods To achieve reliable estimates of p(stop|win) and p(stop|loss), players needed to have at least 5 wins and 5 losses in the data. For the remaining players, the relative likelihoods of stopping were computed as outlined above, and analyzed using a mixed ANOVA with prior outcome (loss vs. win, within-subjects) and involvement level (high vs. low, between-subjects) as independent variables. We then conducted a series of within-group and between-group pairwise comparisons to break down the effects from ANOVA.

We think this analysis approach may offer multiple benefits. First, it directly deals with the probabilities of ending a session after a win versus after a loss, which makes the interpretation of the results more intuitive. Second, the same analysis approach, namely a mixed ANOVA with prior outcome (loss vs. win, within-subjects) and involvement level (high vs. low, between-subjects) as independent variables can now be used for all three facets. Lastly, in the original analysis, we required players to have at least 5 sessions to be included in the analysis. For the new analysis, a less stringent inclusion criterion was used (at least 5 wins and 5 losses; the same criterion was used for the other two facets). As a result, the new analysis included more players than the original analysis. Importantly, the results from the new analysis are largely consistent with those from the original analysis (further highlighting the robustness of our findings).

Minor

1. P2 please use ‘person first language’ i.e. people with gambling problems, not ‘problem gamblers’.

We now use ‘person first language’ throughout the manuscript.

2. Pg 6 line 218 – 230. Rather than endorsing or rejecting the terminology from the gambling operator (‘pathological addictive gambler’ etc), I think this section could be shortened just by introducing the five levels and the binarization.

Thank you for this suggestion. We have now shortened this section by directly referring to the different levels of gambling involvement, without using the terminology of the operator. Please see our reply to Comment 5 by the editor.

3. Table 1 please add units to the parameters, e.g. is the mean and median win in Euros?

Players indeed bet in Euros. We have added units to the parameters in Table 3 (originally Table 1).

4. Not a huge problem but printed in black and white, the two lines in Fig 3 and elsewhere are indistinguishable.

To make sure Fig 3 can be understood even without the color information (e.g., when printed in black and white), we have now added extra information to distinguish the two groups: the means of the two groups are represented by two types of shapes, and the lines connecting the same group also differ. Please see the updated figure in our reply to Comment 6 by the editor above.

Comments by reviewer 2

1. In this manuscript the authors study the phenomenon of “loss chasing” in gambling data from a large dataset obtained from an online gambling company. The experimental setting describe an agent engaged with a repeated decision-making task, where in each trial the agent may choose to continue or halt participation in a gamble that may result a win or a loss. If the agent decides to continue gambling, they also choose whether to change the previous gambled amount (the “stake”). In this framework, loss chasing is the tendency to increase the stakes and probability of choosing to continue gambling specifically in trials that follow losses. In terms of the probability to continue gambling, the authors identify the opposite of loss chasing with an increased likelihood to stop gambling after losing compared to after winning (Fig 3.a). In changing gambled stakes, participants were more likely to change their stake after a loss compared to a win. However, stake change after loss and win were both very rare (2% and 1% to change stake respectively), and the amount of stake changing after loss was centered around zero (Fig 3.b). In testing reaction times, the authors found that participants made quicker decisions following a loss compared to wins. Across these behavioral features, the authors compare the behavior of light and heavy gamblers.

The manuscript seems methodologically sound. Given the importance of better understanding gambling behavior, and the limitation in studying such behavior in the

lab setting, the extensive ecological dataset analyzed here is valuable in unveiling the underlying mechanisms of gambling in its common and pathological forms. My main concern in reviewing the manuscript is a lack of clear message. Having studied the data, what new insights did we gain about gambling behavior? Does the evidence support the notion of loss chasing or does it not? What do the findings suggest on the decision-making mechanisms of gamblers? What do they imply to the vulnerability of gamblers to specific types of games? How would these vulnerabilities be expressed in different types of gambling games? How could the novel understanding from the present study be utilized for the design of interventions in gambling disorders? It is not necessary to address all these questions. Rather, a better distilling of a main message and its implications in some broader context could strengthen the manuscript and its impact, and make it easier to follow. Minor refinements or a reframing of the introduction, and some expansion of the discussion may accomplish this goal.

Thank you for this suggestion. We have now added a new section in the Discussion section, in which we discuss what these results may imply for chasing in real gambling in general. Please see our reply to Comment 1 by the editor.

Major comments
----
2. The main effect of loss chasing is implicated in the tendency to chase, namely continue gambling (possibly with greater stakes), after a loss. As the authors suggest, comparing the groups of high- and low-involvement gamblers should consider each group's winning base-rate, which is higher for the high-involvement groups. This is equivalent to the comparison of two geometrical distributions with a different p ("The probability distribution of the number X of Bernoulli [with probability p] trials needed to get one success" [Wikipedia]). The question of whether participants engage in loss chasing is equivalent to asking what is the probability, given the data, that participants do not in fact have a fixed probability p for stopping, but that p is conditioned on the outcome of the previous trial. Figure 3.a greatly demonstrate this effect. Still, because it was unintuitive for me to mentally compare two geometrical distributions with different p's, it was difficult for me to appreciate the magnitude of this effect in light of a null model which assumes that participants chose to stop gambling regardless of the last outcome. One way to help the reader could be to simulate the null model and position the present data within this simulation. One way to approach this is to simulate a vector of wins and losses according to the empirical distribution of probabilities to win or lose in the studied game. With this simulated vector of wins and losses, consider the probabilities pStop_loss^high, pStop_win^high, pStop_loss^low, pStop_win^low as the simulated probability to stop after a loss or a win in the high and low involvement groups. A null model would assume that pStop_win=pStop_loss= the empirical probability to stop. The distribution of probability of to stop for each of the groups may serve as the null hypothesis which by comparison would allow to appreciate the current data. After simulating this null hypothesis, subsequent simulations may push pStop_win and pStop_loss gradually apart (pStop_loss-pStop_win=0.01, 0.02,…). The resulting sequence of distributions (per each [pStop_loss-pStop_win] difference) may allow the

estimation of: a. what is the most probable p_win and p_loss given the data, b. how likely it is that they are different within each group and c. between groups.

Thank you for this suggestion. We were indeed mainly interested in the probabilities of ending a session after a win (i.e., p(stop|win)) and after a loss (i.e., p(stop|loss)). While our original analysis based on p(win-end) and p(win-overall) allowed us to infer the difference between p(stop|win) and p(stop|loss), this conversion was not very straightforward. We have therefore adjusted the analysis on when to stop, by using p(stop|win) and p(stop|loss) directly. Please see our reply to the major comment 3 by Reviewer 1. Because the updated analysis deals directly with p(stop|win) and p(stop|loss), it also becomes unnecessary to simulate data to show the relationships between p(stop|win) and p(stop|loss) on the one hand, and p(win-end) and p(win-overall), on the other hand.

Minor comments
----
1. The introduction is somewhat repetitive and could be edited to be shorter and more concise. In general, I could easily understand the text, but the manuscript could benefit from further editing and proofreading.

Thank you for this suggestion. We have revised the introduction to reduce the repetition. Furthermore, information that does not appear to be essential (based on some of the comments below from Reviewer 2) has also been removed.

2. It might not be my place to comment on this, but it stood out - Given that one of the perspectives of the manuscript is gambling disorder, it is slightly odd that the first sentence of the introduction frame gambling as a recreational activity that people engage with for pleasure and fun (in the same way that a paper on endangered species may choose to refrain from framing sports hunting as a recreational activity that people engage with for pleasure; even if evidently both claims are true).

We have now revised the first sentence to "Gambling is a popular activity worldwide.", thus without assuming the aim (i.e., for pleasure) for engaging in gambling.

3. “The influential pathways model proposed three distinct pathways from initial 8 gambling exposure to problem gambling [5] While different psychological and biological 9 processes are involved in the distinct pathways, all three …” It is not clear to the reader who is unfamiliar with the pathway model that problem gambling is one type of a pathway. More broadly, if the three different paths are of interest (it seems they are), it would assist the reader if they would be clearly defined here.

We refer to the pathways model because it proposes that loss-chasing is the common step that marks the transition from non-problem to problem gambling, thereby highlighting the centrality of loss-chasing in gambling disorder. The specific pathways themselves were not of interest at that stage. We therefore removed the mentioning of the specific pathways:

One important behavioral phenomenon that is generally considered to mark the transition from non-problem to problem gambling is chasing [5]. 'Chasing', or more commonly loss-chasing, describes a gambler's tendency to continue or intensify gambling to recoup

previous losses. Loss-chasing is widely regarded as a defining feature of gambling disorder [6, 7].

4. line 14: "It is also one of the only signs of disordered gambling that can be directly captured in gambling behavior… The decision to continue gambling despite substantial financial losses is arguably the 38 most prototypical behavioral manifestation of loss-chasing" It is not essentials to your claims, but the reader remains wondering – why is this true? Is this a clinical, mechanistic, or other type of argument?

We included these two statements to highlight the fact that while loss-chasing may be captured in gambling behavior itself, other criteria used in the clinical diagnosis of gambling disorder, such as 'gambles as a way of escaping from problems or of relieving a dysphoric mood (e.g. feelings of helplessness, guilt, anxiety, depression)' cannot be directly captured in gambling behavior. However, we agree that these two statements are not essential, and have therefore removed them in the revised manuscript for the sake of conciseness.

5. Figure 1 has no caption, and I think a caption would assist the reader.

We have now added the general rules of the game in the caption of Fig 2 (originally Fig 1).

6. Figure 1 shows the conversion between points and prizes which for most of the table has a conversion factor of 50 (100 points equal a prize of 20.0), but for the higher amounts of 600 and 1000 the conversions rate changes. I couldn't understand if this is on purpose and of importance (it may be addressed in the text and I just missed it).

Thank you for bringing this to our attention. We went back to the game and double-checked the conversion rates. Indeed, when players win more than 400 points, the conversion rate changes, so that the points are worth more money. This change in conversion rates is included to introduce big wins (i.e., jackpots) into the game. However, for the purpose of the current paper, this feature is not of importance, as we focused on the influence of wins and losses, rather than the exact win or loss amount of each round.

7. It would assist the understanding of the game if several game-strategies could be explained or demonstrated. It seems there are a few degrees of freedom to participants of the game – where to place the columns, when to stop. Is the game based purely on luck (e.g. like a roulette) or does it have some elements of competency (like black jack)? Namely, could the game be played better by a competent player?

Since we did not have data on which dice appeared in each column, and where each column was placed , it is not possible to see whether players used specific strategies (i.e., we only have data on the speed of placing each of the columns). However, *Mystery Arena* indeed contains a 'skill' component, as players can decide where to place each of the 12 columns. As such, this may explain why the high-involvement group overall had a higher winning probability than the low-involvement group. We now explicitly comment on this chance/skill aspect of the game. See our reply to the major comment 2 by Reviewer 1.

8. Also, it wasn't completely clear how are the cost of participating in a game calculated – are they determined per new draw of a dice-column? If so, could the stakes be modified between draws of dices? Does it mean that costs increased with each decision to draw a new column? Or, was participation cost a single amount set prior to a session? If so, why would it be beneficial to stop before the end of a session (before filling all the columns)?

We have now included a section to explain the general rules of the game, and a flowchart to visualize the events within one round of play. Please see our reply to comment 3 by the editor, which is included again below for convenience.

General rules of the game

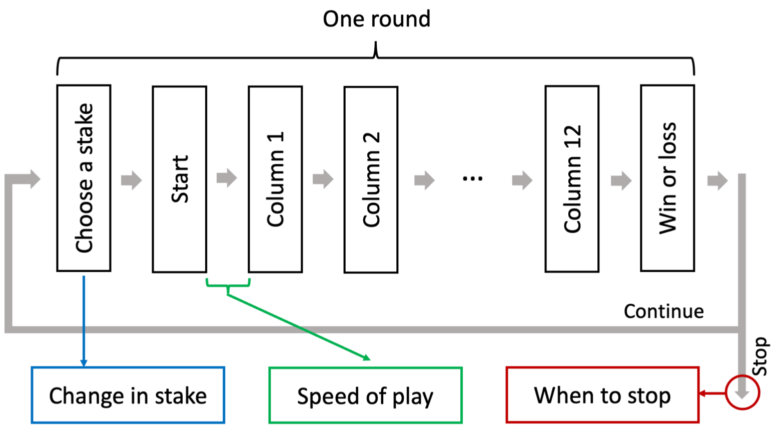

Figure 1. A flowchart showing the events within one round, and which event each expression of loss-chasing is based on.

Before starting a round, players needed to place a stake (Fig 1). The stake was for the whole round, and could not be changed after pressing start. Twelve columns of dice (three dice per column) then appeared one by one. Players needed to put these 12 columns into 4 slots, with 3 columns in each slot. They won points in a slot when a horizontal or a diagonal line of the slot contained the same dice. After all 12 columns had been placed, the points from all 4 slots were added up and converted into monetary prizes (win or loss). After finishing a round, players could continue or end the session.

We hope the new section and the figure clarify the questions raised by Reviewer 2. More specifically, a stake is placed before starting a round, but not within a round. One round consists of placing all 12 columns. Drawing a new column within a round thus does not increase the cost, nor do players need to decide when to stop drawing columns. The 'when to stop' variable thus refers to when players decided to end a session after finishing a round, but not within a round.

9. A related question is how did the game itself operate – were dices randomly drawn at each round with uniform distribution? Or by a different distribution? Was the game reactive (changed dice allocation based on the user choices)?

The dice are randomly drawn each time with equal probabilities, and the game is not reactive to the choices the players make. We now add this information when describing the game interface in detail.

10. This is a matter of taste: Figures 2 displays the distribution of age and gender across the two groups. This figure does not, of course, hinder understanding, but age and gender statistics seem like the least interesting aspect of the data; visualizing any of the rows of table 1 would be, subjectively for me, more relevant to the substance of the manuscript.

Thank you for this comment. Because we added a new figure and two new tables during revision, to reduce the length of the manuscript, we have moved the figure showing the distribution of age and gender to the Supplemental Materials. In the Supplemental Materials, we added a new figure showing the distribution of the total number of sessions and rounds played by both groups. Of the many parameters in Table 3 (originally Table 1), we think these first two may be the most interesting to show, as they directly showed that the high-involvement group gambled much more intensively than the low-involvement group. The figure is shown below. In the main text, we refer readers to the Supplemental Materials for these figures.

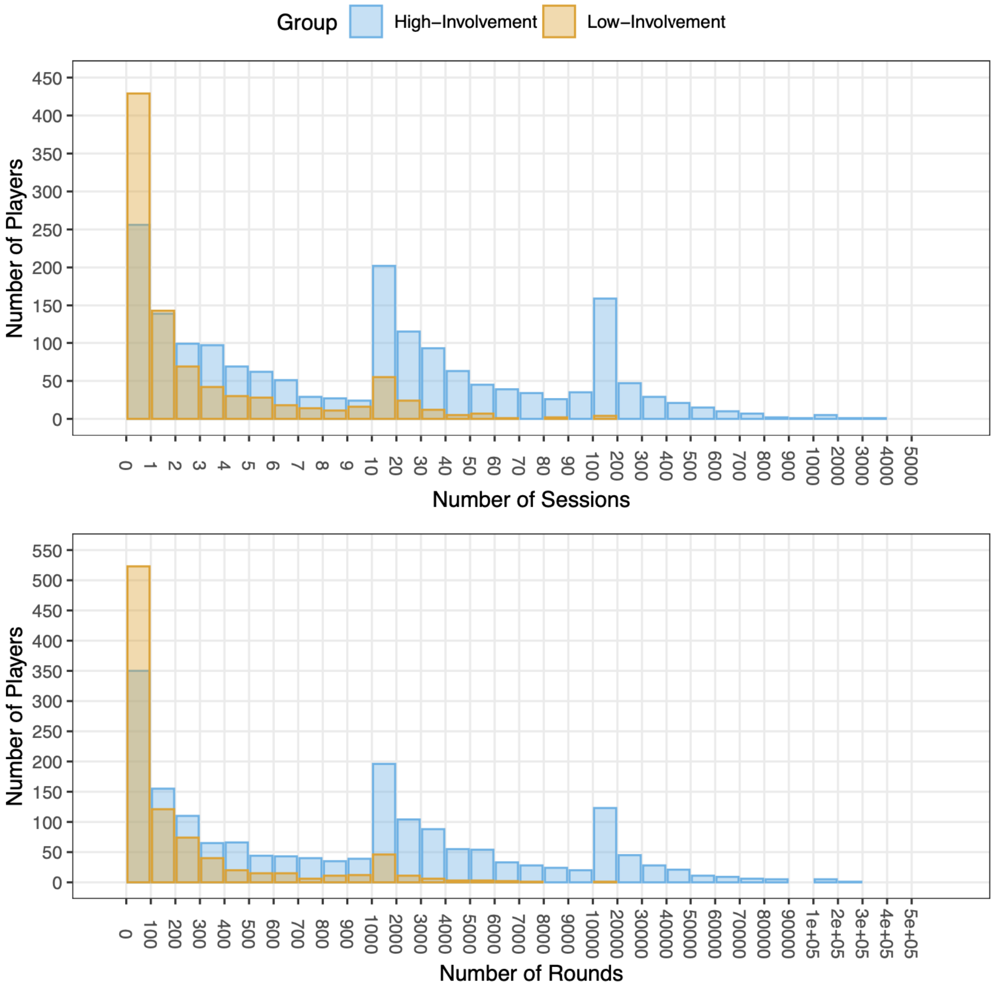

Figure S3. Histograms showing the numbers of players who played certain total numbers of sessions (top) and rounds (bottom). Note that the x axis is transformed. The height of a bar between a and b on the x axis denotes the number of players whose total session or round number is larger than a (> a) but not larger than b (<= b).

11. Although it appears elsewhere, it would be helpful to add the number of participants per group to table 1 (e.g., to remind the reader that there were many more "High" participants compared to "low" ones; thus, explaining how come their "Round number" is so much higher).

We have now added the sample size in each group to Table 3 (originally Table 1). Note that the round and session numbers here stand for the number of sessions and rounds played ***per player***, not all players combined. To facilitate the understanding of the parameters in Table 3, we have now numbered them from (1) till (12) in the table, and used the same numbering in the text and explained each of them.

12. In table 2, 3, 4 the ANOVA's df are identical to the main effects and interaction. I guess this is by design, but it is unintuitive (ANOVA tables for factors A and B are often presented with df |A|-1, |B|-1, (|A|-1)(|B|-1)). A comment addressing this would have helped me understanding the tables.

All ANOVAs used in the current manuscript are 2 by 2 ANOVAs. The degrees of freedom will therefore all be the same, as df1 |A|-1 = 2 - 1 = 1, |B|-1 = 2 -1 = 1, (|A|-1)(|B|-1) = (2 - 1) * (2 -1) = 1, and the same holds for df2. We have now added in the note of all tables that in a 2 by 2 ANOVA, the dfs for all effects are the same.

13. Table 2 – I couldn't understand the label of the last row. Is it "High- vs. Low-Involvement (Difference[Session End, session Overall])" what is "left" and what is "right" here?

Note that the labels in Table 4 (originally Table 2) have been adjusted, because we updated the analysis on when to stop. Each pairwise comparison involves two variables, which we denote as **Comparison (A vs. B)**. The next two rows, **A-mean** and **B-mean**, show the means of the A and B variables, with the standard deviations reported in parentheses. See the note beneath the table.

The last row in the Pairwise comparisons section involves the comparison of difference scores. We now directly specify how the difference score is computed, by using the label **(High-Loss - High-Win) vs. (Low-Loss - Low-Win)**. Thus, for the **High**-Involvement group, the difference between the **Loss** and **Win** condition is computed. Then, for the **Low**-Involvement group, the difference between the **Loss** and **Win** condition is similarly computed. The difference scores are then compared in a pairwise comparison.

Table 4. Statistical analyses on when to stop.

Number of players and rounds included in the analyses in each group									
**Group**	**Player**		**Round (Mean)**		**Round (SD)**		**Round (Min)**	**Round (Max)**	
High-Involvement	1679		6001.9		14176.8		12	200706	
Low-Involvement	651		440.9		959.3		13	13114	
ANOVA									
**Effect**	**df**		**MSE**		**F**		**ges**	**p**	
Involvement Level (High vs. Low)	1, 2328		0.09		5.36		<.001	.021	
Prior Outcome (Loss vs. Win)	1, 2328		0.29		3353.74		.520	<.001	
Interaction	1, 2328		0.29		8.95		.003	.003	
Pairwise comparisons									
**Comparison (A vs. B)**	**A-mean**	**B-mean**	**diff**	**lowerCI**	**upperCI**	**df**	**t**	**p**	**lnBF**	**g**
High-Loss vs. High-Win	1.24 (0.15)	0.17 (0.49)	1.07	1.04	1.10	1678.0	68.5	<.001	1115.02	3.297
Low-Loss vs. Low-Win	1.21 (0.22)	0.25 (0.79)	0.96	0.88	1.04	650.0	24.4	<.001	207.52	1.884
High-Loss vs. Low-Loss	1.24 (0.15)	1.21 (0.22)	0.03	0.01	0.05	898.1	3.0	.007	3.33	0.140
High-Win vs. Low-Win	0.17 (0.49)	0.25 (0.79)	-0.08	-0.14	-0.01	850.1	-2.3	0.027	0.83	0.105
(High-Loss - High-Win) vs. (Low-Loss - Low-Win)	1.07 (0.64)	0.96 (1.01)	0.10	0.02	0.19	860.4	2.5	0.027	1.47	0.114

Note: *ANOVA*: df = degrees of freedom. In a 2 by 2 ANOVA, the dfs for all effects are the same. MSE = mean square of the error. ges = generalized eta squared. *Pairwise comparisons*: Comparison (A vs. B) = the two variables compared in each comparison. A-mean, B-mean = means of the left (A) and the right (B) variable in a comparison, with standard deviations in parentheses. diff = difference between A and B. lowerCI, upperCI = lower and upper boundary of 95% confidence intervals of the difference. df, t, p = degrees of freedom, t value and p value from the Welch's t tests (between-subjects comparisons) or paired-samples t tests (within-subjects comparisons). P values were corrected for multiple comparisons using the Holm-Bonferroni method. lnBF = the natural logarithm of Bayes factors. g = Hedges's average g.

Tables 5 and 6 (statistical analyses on change in stake and speed of play) use the same labels.

14. The Introduction is somewhat confusing in that when I approached the results, I was under the impression that the authors are about to demonstrate loss chasing in its most intuitive form – that gamblers tend to chase (continue gambling) after losses. The fact that the data suggests the opposite is important and interesting; but the expectations build up I had given previous sections left my quite confused. The way I read it, I was expecting to find that losses were followed by increased tendency to stop, and then the respective result is just glossed over ("Overall, p(win-end) was significantly lower than p(win-overall) 350 (Fig 3, Panel (A)). Players were thus more likely to stop after losing than after winning").

In short, the way the introduction is written I was expecting to find that participants have greater likelihood to gamble after losses and it took me some time to understand that what the authors find is the opposite. It would have been easier for me to comprehend the text if the introduction would "prepare" me to this result, or be more clear about the fact that this result is surprising; or more explicit that this findings is inconsistent with loss chasing.

Thank you for this suggestion. We now make it clear at multiple points (including in the introduction) that we indeed did not observe loss-chasing for when to stop. First of all, in the introduction, we explicitly acknowledge that we did not have strong directional predictions *a priori*, and instead focused on characterizing the presence or absence of loss-chasing in each of the three facets (see our reply to Comment 2 by the editor). Second, in the statistical analyses section, as a preview of the results, we added a column in Table 2 showing that we did not observe loss-chasing for the first two facets (i.e., when to stop and change in stake), but only for the third facet (i.e., speed of play).

When describing the results on when to stop, we also explicitly note that we observed win-chasing, instead of loss-chasing with this facet.

Players were more likely to stop after a loss than after a win (Fig 3, Panel (A)). This result is thus opposite to the prediction of loss-chasing (Table 2), but consistent with the results by Leino and colleagues on EGMs players [9]. Players in the current data set thus did not seem to chase losses; instead, they chased wins, by being more likely to continue playing after winning.

In the general discussion, we again start with a summary of the direction of the effect observed for each facet.

Overall, both high- and low-involvement players were less likely to stop after a win than after a loss (i.e., win-chasing in when to stop). Furthermore, both groups increased the stake amount more after a win than after a loss (i.e., win-chasing in stake amount). For the speed of play, both groups responded more quickly after a loss than after a win (i.e., loss-chasing in speed of play), with a smaller effect in high-involvement players.

By clearly describing the inconsistency of the results with loss-chasing, we think it should be clear to the readers that we observed the opposite of loss-chasing. In the current data set, players were less likely to stop after a win than after a loss.
