# Supplementary material for: Winning and losing in online gambling: Effects on within-session chasing
Source: PLoS One. 2022 Aug 18;17(8):e0273359. doi: 10.1371/journal.pone.0273359 (PMC9387854; doi:10.1371/journal.pone.0273359)
Supplement: Attachment — Submitted filename: responses.pdf [file pone.0273359.s003.pdf]

Dear editor,

Thank you again for the opportunity to revise our manuscript. In this letter, we list the comments by the two reviewers **in bold**, and explain how we have addressed each comment by Reviewer #2. Texts from the revised manuscript are shown in green. In the pdf file of the revised manuscript, we have highlighted the changes.

Kind regards,
Zhang Chen

Reviewer #1: The authors have been very attentive to the referee comments. This is a sophisticated piece of research: they are using high-resolution behavioural gambling data from a real online gambling platform, in a large dataset (10 million bets), looking at three distinct behavioural expressions that each require careful operationalizing. The value of the paper is in the contrast of the three expressions of chasing. They have done an excellent job of conveying their objectives and analysis pipeline as clearly as possible.

We would like to thank Reviewer #1 for these encouraging words, and for his constructive comments on our manuscript.

Reviewer #2: I was very happy to receive the updated manuscript. The revised version substantially improves on its previous. The Introduction is more concise, the Methods easier to follow, and the discussion better explains the multifaceted nature of loss chasing. In particular, I found table 1 to be helpful in shaping the reader's expectations from the findings. Similarly, the grouping of all findings into a single figure (Fig. 3) is helpful. I thank the authors for the comprehensive point-by-point responses to my previous comments.

We would also like to thank Reviewer #2 for the constructive comments, which have greatly improved the quality and clarity of our manuscript.

I have only minor comments:

1. Line 153 - "Note that players could also start a new round before the 'PLAY AGAIN' message appeared" – I wasn't sure what to make of this comment. If I understand correctly, games that concluded in a win were followed by a pleasant animation that participants may have chosen to savor. Games that were concluded by a loss were not. The comparison of timing between [win, something nice, continue?] and [lose, continue?] suggests then that the choice to continue to an additional game would be – by the construction of the game – quicker after losses, as indeed indicated by the results (e.g., Fig 3.D). Is this comment offered as a limitation to the finding of quicker RT after a loss? If so, it can potentially be framed as such more explicitly. Also, a comparison of the animation time and the difference between wins and losses reaction time may shed light on the role of the animation in generating this effect (if the animation is 1 second and the mean difference between wins and losses RT is 5 seconds then the effects of the animation are potentially of lesser concern).

Alternatively, does this comment just explains the actual structure of the game and has no relation to RT?

The feedback event is indeed generally longer after a win than after a loss. For this reason, we did not use the speed of initiating a new game as the indicator for the speed of play, but rather the interval between starting a new game till inserting the first column in a new game. The RT that we used is therefore not confounded by the factors mentioned by the reviewer, as we described in the data analysis section (line 271):

The speed of starting a round in the behavioral tracking data is the duration from when players placed the last column in the previous round till when they started the current round (Table 1). This duration included the different feedback events after winning and losing, and the time spent on adjusting the stake. Due to these confounding factors, we used the response time (RT) of placing the first column as the behavioral indicator of speed of play instead. This RT is from when players started the current round till when they put the first column in one of the 4 slots, and thus is not confounded by the factors mentioned above.

To make sure that readers clearly understand which RT indicator was used, when explaining the general procedure, we now immediately add a clarification in the revised manuscript (line 153):

Due to the differences in the feedback after wins and losses, and the flexibility in when to start a new round, we did not use the speed of starting a new round as the indicator for the speed of play. Instead, we used the interval between starting a new round till placing the first column in the new round (see Fig 1), which is not confounded by the factors discussed above.

2. I do not understand participants inclusion criterion. Line (236): “players needed to have at least 5 wins and 5 losses in the data”, line (255) “players again needed to have at least 5 rounds following a win and 5 rounds following a loss” – the ‘again’ seems to imply this is the same criterion but I’m not sure that it is: a player may have a single loss followed by 6 wins, satisfying the second condition but not the first.

The reviewer is correct in noting that these two inclusion criteria are not the same. To avoid misunderstanding, we have deleted the word ‘again’ on line 255.

3. Fig 3.a axis labels – The use of relative likelihoods of ending a session is well explained. However, I don’t know “relative likelihood” as a standard term. Due to the importance of the panel, and the possibly not so intuitive units of that panel, it would have been easier for me if “relative likelihood” was also described in the figure caption. Something like “relative likelihoods are the conditional stopping probabilities given a win or a loss, normalized per-participant by the overall probability to stop, e.g. for losses P(stop|loss)/P(stop-overall)”.

Thank you for this suggestion. We have now added an explanation on relative likelihoods in the caption of Figure 3.

For panel (A), relative likelihoods are the conditional probabilities of stopping after a loss and

after a win, normalized per player by the overall probability to stop, e.g. $p(stop|loss)/p(stop - overall)$ and $p(stop|win)/p(stop - overall)$.
